# Children’s experiences and responses towards an intervention for psychological preparation for radiotherapy

**DOI:** 10.1186/s13014-017-0942-5

**Published:** 2018-01-22

**Authors:** Gunn Engvall, Viveca Lindh, Tara Mullaney, Tufve Nyholm, Jack Lindh, Charlotte Ångström-Brännström

**Affiliations:** 1 Department of Women’s and Children’s Health, Uppsala University, Uppsala, Sweden; 2 Department of Nursing, Umea University, Umea, Sweden; 3 Veryday, Stockholm, Sweden; 4 Department of Radiation Sciences, Umea University, Umea, Sweden

**Keywords:** Psychological preparation, Distraction, Radiotherapy, Childhood cancer, Children’s experiences

## Abstract

**Background:**

Children can experience distress when undergoing radiotherapy as a reaction to being scared of and unfamiliar with the procedure. The aim was to evaluate children’s experiences and responses towards an intervention for psychological preparation for radiotherapy.

**Methods:**

A case control design with qualitative content analysis of semi-structured interviews and statistical analysis of anxiety ratings were used for evaluating a strategy for psychological preparation and distraction. Fifty-seven children aged 2 to 18 years and their parents participated – 30 children in the baseline group and 27 in the intervention group. Child interviews were performed and the child and their parents rated the child’s anxiety.

**Results:**

The intervention was most appropriate for the younger children, who enjoyed the digital story, the stuffed animal and training with their parents. There were some technical problems and the digital story was not detailed enough to fit exactly with various cancer diagnoses. Children described suggestions for improvement of the intervention. The ratings of the child’s anxiety during radiation treatment showed no differences between the baseline group and the intervention group.

**Conclusions:**

The children of all the age groups experienced their interventions as positive. The strength of the intervention was that it encouraged interaction within the family and provided an opportunity for siblings and peers to take part in what the child was going through. Future research on children’s experiences to interventions should be encouraged. The intervention and the technical solutions could improve by further development.

**Trial registration:**

The study design was structured as an un-matched case-control study, baseline group vs. intervention group. Trial registration: ClinicalTrials.gov NCT02993978, Protocol Record 2012–113-31 M. Retrospectively registered - 21 November 2016.

## Background

Children with cancer can be treated with radiotherapy (RT) solely or combined with chemotherapy and/or surgery. In Sweden about 300 children 0–18 years annually are diagnosed with cancer [1] and according to data from the Swedish Radtox registry, approximately 80–100 children undergo RT annually. Although RT is painless and noninvasive, children can experience distress as a reaction to being scared of and unfamiliar with the procedure, meeting with new hospital staff, being separated from parents, and the sounds from the unfamiliar equipment [2, 3]. There are considerable demands on the child to stay motionless during RT for optimal results and safety reasons. Thus, treatments with repeated sedations, drug use and general anesthesia are often required for the youngest children i.e. the preschool children, making each treatment expensive and time consuming as well as affecting the child’s daily life [3–5].

There are few studies describing children’s experiences with RT. Children with brain tumors, 4–16 years old, who were undergoing RT experienced boredom and discomfort, they missed school and peers, activities they usually did, and appreciated having a parent close by [6]. Furthermore friendly staff who listened and explained helped them through treatment [6].

Some intervention studies aiming to reduce distress and anxiety and the need for sedation and anesthesia among children undergoing RT have been performed. An intervention was used to minimize children’s anxiety and children aged 3.5–6 years old were given explanations and instructions about RT, made visits to the radiation unit, and an intervention by an arts therapist was carried out [3]. The result shows that only 5 of 55 children in total needed anesthesia when being treated. The authors conclude that it is important for staff to be flexible, open to improvisation and to be aware of each child’s and family’s specific needs and capacities [3]. Play preparation for children 2–5 years old undergoing RT can minimized the need for sedation [7].

An audiovisual interventions was implemented to avoid anesthesia with children undergoing RT [5]. The choice of the intervention (movie, DVD or microphone) was made by the child and the result showed that 22 of the 24 children aged 2–6 years who received the intervention successfully had part of or all their radiation without anesthesia [5]. A psychoeducational intervention including a play program and interactive support to get familiar with the staff, equipment and procedure of the RT was used [4].

The efficacy of an interactive-educational intervention in reducing pediatric distress and parental anxiety associated with radiotherapy-related procedures was examined [8]. The findings in the intervention group showed that the children were less frightened, parents experienced significantly greater reductions in stress and family distress was reduced [8]. Further, Play therapy sessions in combination with audiovisual aids – for example, cartoons – for children younger than 7 years, before the start of treatment with external beam radiation therapy was implemented, and the need for sedation was reduced [9]. It is well known that visual preparation is appropriate for children [5, 8, 9].

The literature review showed that several interventions in pediatric radiotherapy could decrease anxiety and distress in children going through RT. The interventions were mostly evaluated in terms of parental anxiety and reduced need for sedation and anesthesia. However, no studies were found where the children themselves participated in the evaluation of the interventions. We also found no intervention studies where the child was given the opportunity to train together with their family at home before RT started. Children’s overall experiences during RT treatment have been described previously [6, 10], though there are few studies where children describe their experiences of specific interventions used during the RT, and none in a Scandinavian context. In order to create a cohesive strategy for psychological preparation and distraction we worked with design researchers at the Umeå Institute of Design using a Human Centered Design (HCD) approach and evaluated the intervention by using children’s self-reports and child interviews.

The aim of this study was to evaluate children’s experiences and responses towards an intervention for psychological preparation for radiotherapy.

## Methods

### Study design

The study was conducted during a baseline period, September 2012 to January 2014, followed by an implementation and evaluation period of a designed intervention for psychological preparation and distraction of children during radiotherapy, February 2014 to June 2015. The study design was structured as an un-matched case-control study (baseline group vs. intervention group), ClinicalTrials.gov Protocol Record 2012–113-31 M. Qualitative content analysis of semi-structured interviews and statistical analysis of anxiety ratings were used for evaluating the strategy for psychological preparation and distraction The study was performed in three out of six Pediatric RT centers for treatment of children with cancer in Sweden, i.e. the Departments of Radiation at Uppsala University Hospital, Karolinska University Hospital, Stockholm and Umeå University Hospital, Sweden.

Students at the Institute of Design at Umeå University, Sweden developed the intervention in 2013 during the baseline period. Clinical routines for preparation and distraction of children remained unchanged during the baseline period and differed somewhat between centers [10].

### Sample

Fifty-seven children from 2 to 18 years old diagnosed with cancer and admitted to RT at one of the three pediatric oncology centers and their parents were included (Table 1). The sample was stratified according to age and gender of the children and to represent the three pediatric oncology centers in the baseline group and the intervention group. Part of the sample was also stratified for interviews after RT in respective groups. To be eligible for the intervention the child should be scheduled for CT and fixation at least 5 days after study inclusion in order to have time to go through and learn the preparation material.

**Table 1:** Participants in the baseline group and the intervention group

Participants	Total *N*	Baseline N (Mean age ± SD)	Intervention N (Mean age ± SD)	Interviews N total/baseline/intervention
Children	*N* = 57	*N* = 30 (9 ± 4.5)	*N* = 27 (10 ± 5.1)	33/13/20
		Girls *N* = 15 (8.8 ± 4.7)	Girls *N* = 13 (9.8 ± 4.8)	
		Boys *N* = 15 (9 ± 4.5)	Boys *N* = 14 (10.6 ± 5.4)	
Mothers	*N* = 56	*N* = 29 (42.2 ± 6.3)	*N* = 27 (41.8 ± 6.1)	
Fathers	*N* = 57	*N* = 30 (42,2 ± 6.3)	*N* = 27 (43,7 ± 5.2)	

All children received active treatment according to international or national study protocols used for the different diagnoses, or if protocols were unavailable, according to national recommendations/guidelines.

### Data collection/tools

#### The intervention

**Fig. 1:**
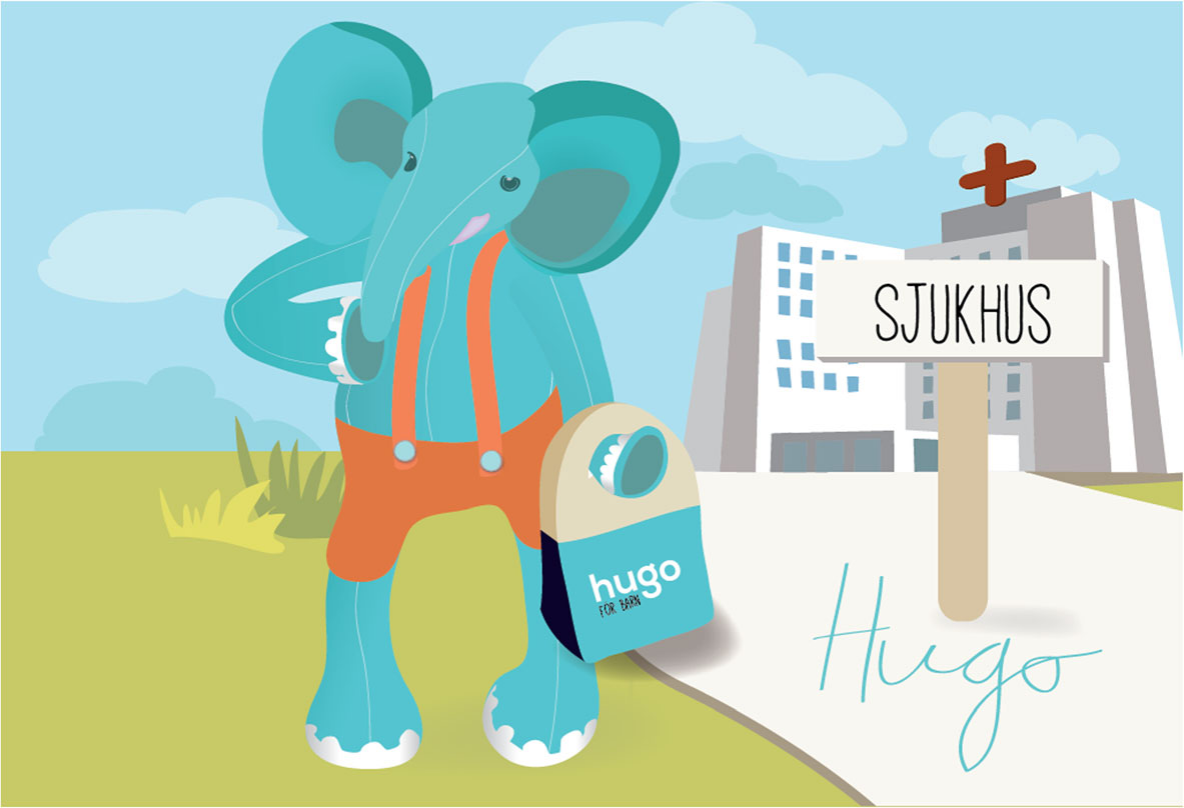
Hugo for kids

Students from Umeå Institute of Design were given a course assignment to create technical and educational tools that could help both children (aged 2–18) and their parents to prepare for and cope with RT. They used an HCD process with a quick ethnographic method to develop the intervention [11]. The resulting preparatory interventions were “HUGO for Kids” (Fig. 1) for children aged 2–12, and “HUGO for Teens” for children aged 12–18 [12]. It was a preparatory kit, including age-adjusted information on tablets, gift of a stuffed toy or a pair of headphones, a parent booklet, and toy models of the computed tomography and RT machines was included [13]. The applications were developed in Swedish and English in order to be useful for the majority of the patients at the participating RT centers. The child and the parent(s) were introduced to the HUGO material by a nurse at the RT department at least 5 days before the start of the RT procedure, thus giving them enough time to prepare at home before RT start.

#### Measurement of anxiety

The children aged 3–10 years rated their anxiety with the faces affective scale (FAS) [14] and the children and adolescents older than 10 years with a visual analogue scale for anxiety (VAS-A) [15]. The parents rated their child’s anxiety (VAS-A) at four evaluation points: (1) at inclusion in the study after RT decision, (2) at the CT and fixation before RT, (3) at the start of RT and (4) when the RT was completed. All children and the parents were requested to respond to the one question “How anxious do you feel right now?” by marking a point along a line (0–10 cm) on the VAS-A scale or pointing on one of the faces on the FAS-scale corresponding to “Not anxious at all” (score 0) up to “Worst anxiety imaginable” (score 10).

#### Interviews

Semi-structured child interviews (*n* = 20, children aged 5–17) were performed by two of the authors (GE and CÅB). Parents were present at face-to-face interviews, and informed to not participate in the conversation. The interviews were performed either in the hospitals (*n* = 17) or by phone (*n* = 3). The interview guide invited the children to talk freely about their experiences of the RT and follow-up questions were posed. Specific questions were posed about the designed intervention in the intervention group. To enhance communication, GE and CÅB asked the children to do a drawing about RT [16]. They decided themselves what they wanted to depict in their drawings and participation was optional. During or after the interview, GE and CÅB asked each child to talk about the drawing to understand what the child meant to convey [17]. The children talked about their experiences with RT.

### Data analysis

#### Statistical analyses

SPSS (IBM SPSS Statistics Data Editor Version 23) software was used for the statistical calculations. Participant demographics were presented with descriptive statistics, group differences between the baseline group and the intervention group were calculated using the Mann–Whitney U test, and for paired comparisons before and after RT the Wilcoxon Signed Rank test was used. Due to logistical reasons there were missing FAS/ VAS-A ratings. The number of completed ratings is shown as N in Tables 2 and 3.

**Table 2:** Children’s ratings of anxiety, (FAS/ VAS-A 0–10) in the baseline group and the intervention group

	Baseline		Intervention	
FAS/VAS – A (0–10)	*N* mean (±SD)	Median (Range)	*N* mean (±SD)	Median (Range)
Study start Evaluation 1	*N* = 15 3.5 (±2.8)	3.0 (0–9)	*N* = 18 3.4 (±2.6)	3.0 (1–10)
Fixation and CT Evaluation 2	*N* = 12) 3.0 (±2.5)	2.0 (0–9)	*N* = 20 3.3 (±2.2	3.0 (0–8)
Start of RT Evaluation 3	*N* = 17 3.4 (±2.4)	3.0 (0–8)	*N* = 23 2.5 (±3.1)	1.7 (0–10)
End of RT Evaluation 4	*N* = 22 2.3 (±2.0)	1.85 (0–9)	*N* = 23 2.5 (±3.1)	1.7 (0–10)

**Table 3:** Parent’s ratings of their child’s anxiety, (VAS-A 0–10) in the baseline group and the intervention group

	Baseline		Intervention	
	*N* VAS-A mean (±SD)	VAS-A median (Range)	*N* VAS-A mean (±SD)	VAS-A median (Range)
Study start Evaluation 1	*N* = 29 4.1 (±2.7)	4.0 (0–10)	*N* = 33 4.6 (±2.4)	4.0 (0–10)
Fixation and CT Evaluation 2	*N* = 26 4.3 (±2.3)	4.0 (0–10)	*N* = 36 4.1 (±2.4)	4.0 (0–10)
Start of RT Evaluation 3	*N* = 33 4.3 (±2.7)	4.0 (0–10)	*N* = 38 3.6 (±2.6)	3.0 (1–8)
End of RT Evaluation 4	*N* = 36 2.6 (±2.4)	2.0 (0–9)	*N* = 33 2.7 (±3.0)	1.0 (0–10)

#### Qualitative analysis

In the qualitative analysis deductive and inductive content analyses were combined [18]. First, all interviews with the children were read through to get a sense of the content and the interviews with the younger and older children were sorted and analyzed separately. The first phase of the analysis was deductive and built on previous reported findings at baseline [10]. These findings were used as a starting point. According to Elo and Kyngas deductive analysis can be used when the structure of the analysis is made on basis of previous knowledge [18]. Hereby, inductively built concepts can be complemented and further developed [18, 19]. Statements from each interview concerning children’s experiences of undergoing RT derived from the earlier study [10] were identified and grouped.

In the second phase an inductive content analysis was performed on the remaining interview data [20]. The interview text was divided into meaning units, condensed, coded, compared and discussed among the authors (VL, GE, CÅB). Thereafter, the data from the interviews with younger and older children were compared and discussed, in order to find out whether there were any differences or similarities. The authors discussed and reflected on the subcategories and categories, and the findings were formulated, resulting in subcategories and categories. Quotations from the transcribed text are shown in the findings. Lastly, the authors looked at the drawings in relation to each child’s interview and his or her conversation with GE and CÅB about the drawing. Nine children in the group younger children did drawings. The drawings were understood on the basis of what the children had described [17] and were sorted to illustrate the categories/subcategories.

### Ethical considerations

Parents were given written and oral information about the study when their child was admitted to RT treatment and parents’ written consent and consent on behalf of their child was obtained. The children were given age-adjusted written and oral information and were asked if they would like to participate. Children under the age of 15 gave their oral assent and children older than 15 years gave written consent to participate. The study was approved by The Regional Ethic Review Board, Umeå, Sweden (Ref. no. 2012–113 31 M).

## Results

### Participants in baseline versus intervention group

There were no significant differences between families concerning age, education, work situation (extent), number and age of siblings and whether the child was living with both or one parent.

The children in the baseline group were diagnosed with acute lymphoblastic leukemia (ALL) (*n* = 1), different central nervous system (CNS) tumors (*n* = 14), sarcomas (*n* = 6), neuroblastomas (*n* = 5), Hodgkin’s disease (*n* = 3) and Wilms’ tumor (*n* = 1). The children in the intervention group were diagnosed with different CNS tumors (*n* = 13), sarcomas (*n* = 6), neuroblastomas (*n* = 4), Wilms’ tumor (*n* = 3) and Hodgkin’s disease (*n* = 1). There were no statistical differences in the number of children who received chemotherapy treatment and/or surgery before and during RT between groups.

### Anxiety rates- children/parents

For the children’s FAS/VAS-A ratings there were no significant differences between baseline group and intervention group at any of the evaluation points. Both groups of children rated FAS/ VAS-A lower at the end of RT as compared to the study start, though this was only significant when calculated on the total sample (*p* = 0.014), (Table 2).

For the parents ratings of the child’s anxiety there were no significant differences between groups at any of the four evaluation points (Mann–Whitney U test). In the paired comparisons (Related samples Wilcoxon Signed Rank test) VAS-A was shown to be significantly lower in the intervention group at evaluation point 4 as compared to evaluation point 1 (*p* = 0.001), (Table 3).

Five children in the baseline group and five children in the intervention group received anesthesia for the RT. There were no significant differences between groups (Chi-Square test) in the number of children who did or did not have anesthesia. The children who were anesthetized had a median age of 3 years – min. 2 and max. 8 in the baseline group and min. 2 and max. 6 in the intervention group.

### Children’s interviews

The interviews lasted between 7 and 30 min, were tape-recorded and then transcribed verbatim.

The content from the present interviews was in line with the findings from the interviews in the baseline group – see Table 4. The children described: Positive and negative experiences with hospital stays and practical arrangements; Age-appropriate information, communication, and guidance to various degrees; Struggle with emotions; and Use of distraction and other suitable coping strategies. In the present interviews the children did not describe any experiences with olfactory and light sensations. At baseline the younger children did not report that they used problem-solving activities, but one girl described in the present interviews that she chose special clothing to wear so as not to have trouble during RT as she had at the first session. Furthermore, in the baseline group the younger children did not describe physical and psychological problems but this occurred in the intervention group.

**Table 4:** Categories and subcategories in the baseline group (B) and in the intervention group (I), for children 5–10 and 11–17 years (y)

Category	Subcategory	B	B	I	I
		5–10 y	11–15 y	5–10 y	11–17 y
Positive and negative experiences with hospital stays and practical arrangements	Appreciating activities, being bored, and disliking waiting time	x	x	x	x
	Being together with, or missing siblings and peers	x	x	x	x
Age-appropriate information, communication, and guidance to various degrees	Having/lacking/missing information and communication about what is going to happen	x	x	x	x
	Having an exploratory visit and meeting with the staff at the radiotherapy ward	x	x	x	x
Struggle with emotions	Being afraid and feeling anxiety	x	x	x	x
	Disliking and accepting the mask, the dot tattoo, and the machine	x	x	x	x
	Finding the right position and remaining motionless	x	x	x	x
	Disliking the sensations	–	x	–	–
	Suffering physical and psychological problems to various extents	–	x	x	x
	Appreciating small gift	x	–	x	–
Use of distraction and other suitable coping strategies	Using a suitable media distraction	x	x	x	x
	Using problem-solving activities	–	x	x	x
	Using strategies to deal with emotions	x	x	x	x
	Wanting parents close by before, during, and after treatment	x	–	x	–
	Seeking support from parents, staff and peers	–	x	–	x

Experiences of using the material from the intervention revealed three categories of result: Positive and negative experiences of the tablet; Positive experiences of stuffed toys and the CT and RT models; and Suggestions for improvement of the intervention (Table 5). The categories and subcategories are described and presented with quotations from the interviews with code-number, Girl (G) /Boy (B) and age in years (y).

**Table 5:** Categories and subcategories in the intervention group for children 5–10 and 11–17 years (y)

Category	Subcategory	5–10 y	11–17 y
Positive and negative experiences of the tablet	Watching and sharing the digital story about Hugo	x	–
	Looking at and using the information and suggestions for preparation	x	x
	Using material for distractions during the procedure and for amusement	x	x
	Counting down by placing stickers each time on the tablet cover	x	–
Positive experiences of the stuffed toys and the CT/RT models	Appreciating the stuffed toy Hugo to play with	x	–
	Appreciating the RT and CT models to play with	x	–
Suggestions for improvements of the intervention	Desire for adapted and interactive information and solutions	x	x

#### Positive and negative experiences of using the tablet

##### Watching and sharing the digital story about Hugo

**Fig. 2:**
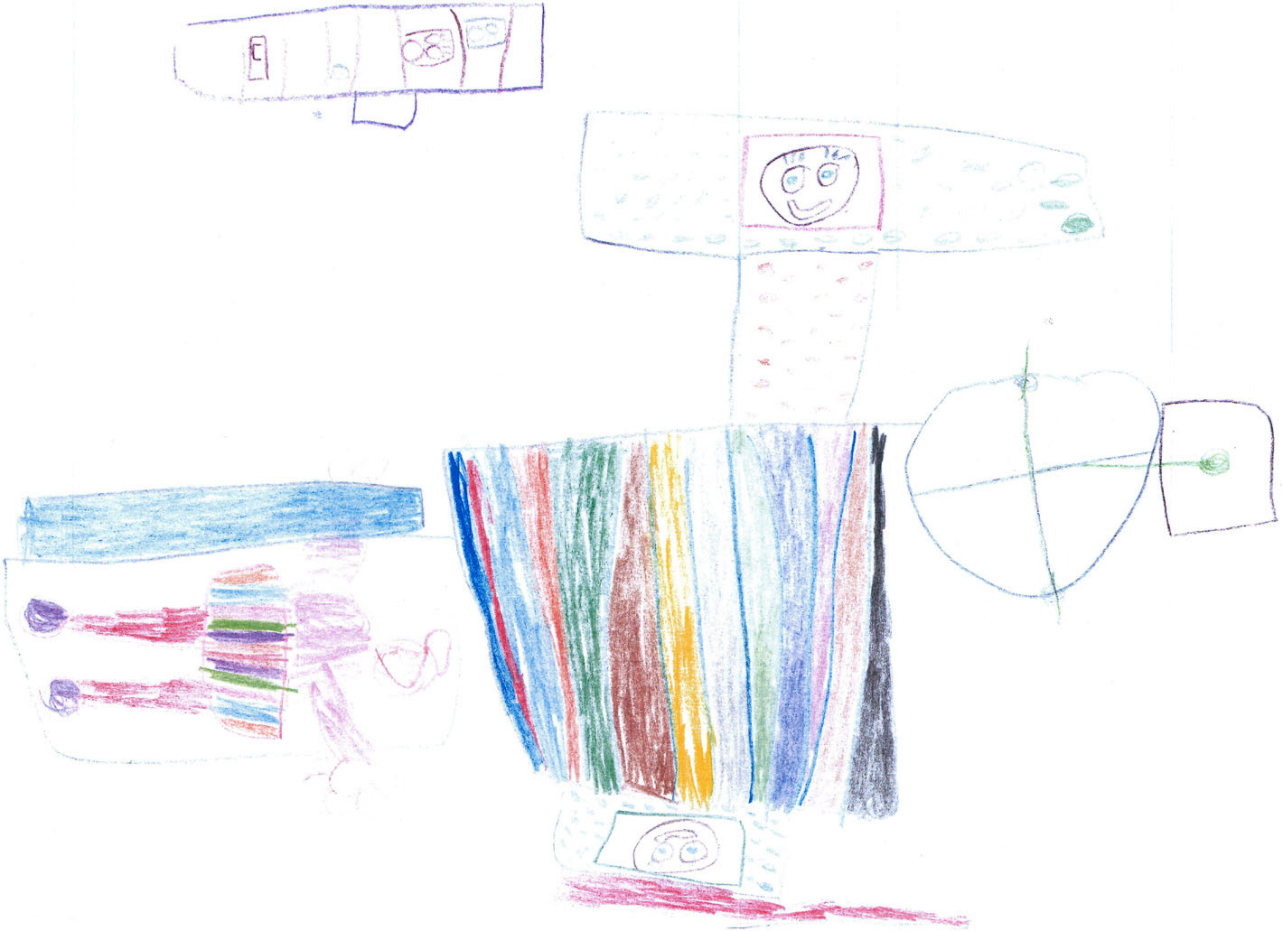
Drawing made by a girl, 6 years old, waiting for the RT to start. The mask is on the shelf to the left, and she has a cushion under her knees

Younger children described how they prepared before undergoing RT, often together with family members, by looking at the story about Hugo, or by looking at pictures in the tablet:

*“Then you get a mask, then a pillow under your legs and you have to lie very still*… *then it’s ok”* (0211 G6y). Figure 2 is an illustration of a child in the treatment room.

The children also described difficulties with the technique: *“Yes. Though the tablet, it wants to, it almost works, well it doesn’t work. Yeah, it doesn’t work, does it? It did work in the beginning, like when we watched Hugo about radiotherapy”* (0318 B6y).

##### Looking at and using the information and suggestions for preparation

Children described that they had used the tablet and the information more or less. Some children had their own handheld devices and were not interested in the tablet, but others described using the tablet often. One of the younger children and her parent described using the propositions for preparation from the tablet such as listening to the noise from the machine when the child trained at the kitchen table to lie motionless. To lie motionless can be very difficult, especially for the youngest children. From the beginning, sedation can be required, but some children later managed the RT without sedation.

The older children did not use the tablet to any significant extent to read the information. Some of them looked briefly at the information, while others did not. Many of the them preferred to use their own phone, tablet or computer, to search for and read information about RT on the Internet: *“I mean, you might feel that you could have one of those tablets, but I might as well have my own cell phone... it feels like it’s a bit too big to walk around with the tablet.”* (0314 B17y). However, they said that their parents and siblings were interested in using the tablet to read and make use of the information: *“It was mostly my big sister who used the tablet.”* (0307 G12y).

##### Using material for distraction during the procedure and for amusement

**Fig. 3:**
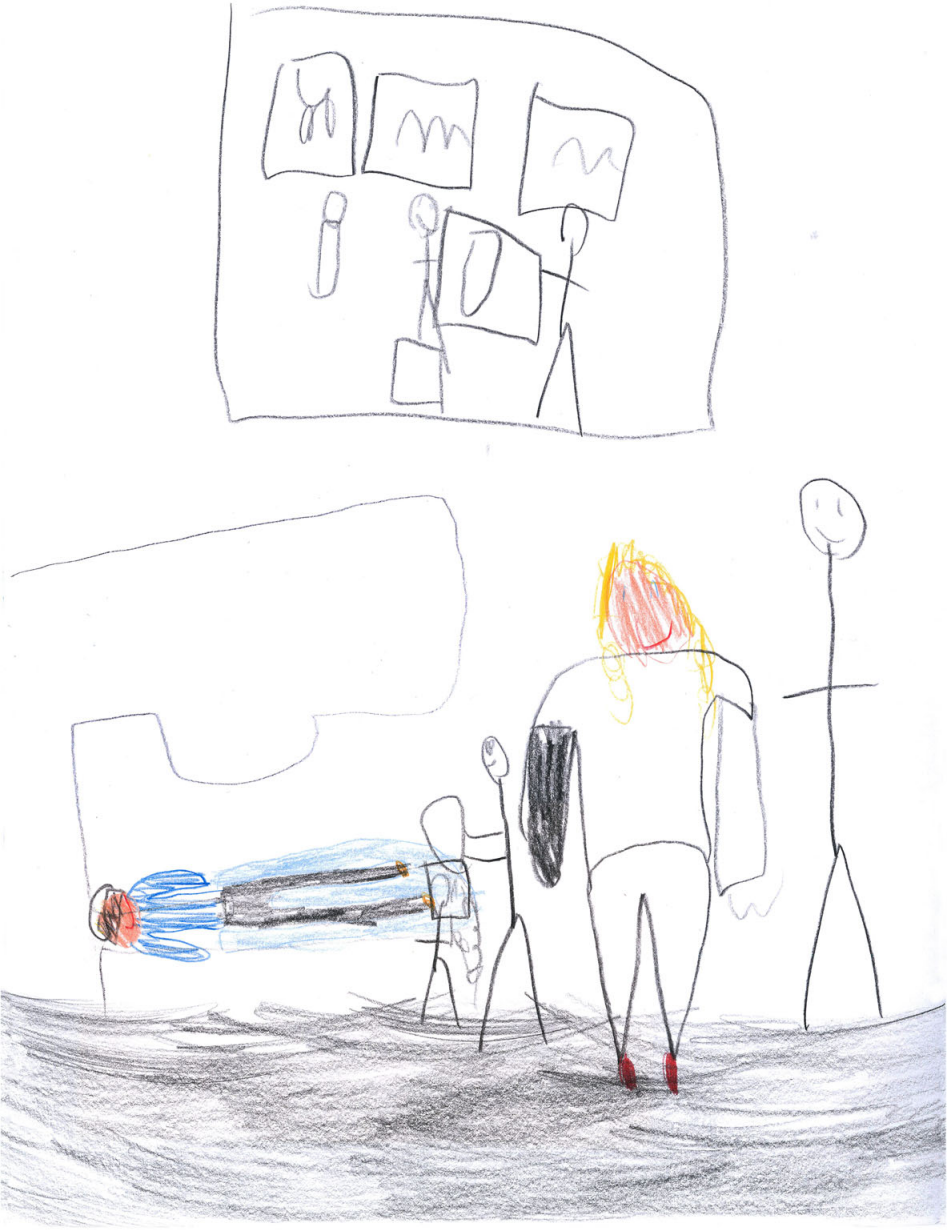
Drawing made by a girl, 10 years old, laying down waiting to hear the music she chose during RT. The staff member in treatment room is preparing the tablet

It was possible to use the tablet during the RT: *“If you’re a child then you have to wear a pair of glasses. So you get to watch things like movies and I usually check out the music videos. And when there’s a good song I start singing along pretty loudly.”* (0111 G9y). Children described using the tablet in different ways: *“I got to download my own fun games. We watched some videos.”* (0327 B9y). Figure 3 is an illustration of listening to music by using the tablet.

Many of the older children described being well informed and prepared for RT and they knew what to expect: *“Well, I wouldn’t say that it was easy, but it works well and it’s convenient.”* (0222 B17y). Another child expressed: *“Radiotherapy hasn’t been hard ... but I think that picture on the ceiling (scene of the moon) is pretty good.”* (0114 B17y). Some children used the tablet to listen to music during the RT, while others were thinking about “*other things*” (0307G 12y). The tablet was used to surf on the Internet, looking at films and play games for amusement.

##### Counting down by placing stickers each time on the tablet cover

Younger children described getting a gift each time they underwent RT*.* They could put the stickers on the tablet cover and counted how many times that was done: *“Then you got stickers every time you ran it – I really liked that.”* (0326 G9y). “*It’s a tablet… how many I’ve had... I’ve had twenty-six. You can see how many balls that is. I only have footballs”* (0318 B6y).

#### Positive experiences of the stuffed toys and the CT/RT models

##### Appreciating the stuffed toy Hugo to play with

Younger children described playing with the stuffed toy Hugo: *“I got a stuffed animal named Hugo. He has cancer too (laughs). He has cancer too and he’s had radiotherapy”* (0111 G9y).

##### Appreciating the RT and CT models to play with

The RT and CT models were used by the younger children, their siblings and friends: *“And I gave radiotherapy to some other stuffed animals that were there too... they turned out great”* (0319 G6y).

#### Suggestions for improvements to the intervention

##### Desire for adapted and interactive information and solutions

Some of the younger children described that the Hugo story and reality did not match: *“They said that you wouldn’t feel anything … and they don’t send out the kinds of rays like they did in the Hugo movie … that’s why I didn’t really like the movie”* [frightened of the radiation from the Hugo story] (0319 G6y). The older children suggested that the information on the tablet could be made more fun: *“It was just boring”* [going through the material on the tablet] (0322 B11y) and that the information could be presented as an animated film. Some children in the older group suggested *“something interactive”* (0306 G14y), *“something to press so something happens”*. Another suggestion from the older children was to develop an app for the phone. *“I don’t think it* [the tablet] *is completely necessary, if you want my opinion … maybe for younger children, but I think they also have phones and things… so maybe it would be better to have something for the phone?*” (0317 B17y).

## Discussion

The aim of this study was to evaluate children’s experiences and responses towards an intervention for psychological preparation for radiotherapy. Main findings were that the psychological intervention was described in the interviews as most useful for the younger children. Findings did not reveal decreased anxiety in the intervention group as measured quantitatively. The intervention cultured interaction within the family system and with peers about the current situation for the child going through RT. In the analyses of the interviews the same main categories occurred after the intervention as for the baseline group [10], revealing the same pattern of positive and negative experiences, age-appropriate information to various degrees, struggle with emotions and use of coping strategies. A few subcategories occurred differently. *Disliking the sensation* did not occur in the intervention group, probably caused by the fact that such side effects are rare. The subcategory *Suffering of physical and psychological problems to various extents* did not appear in the base line group for children aged 5–10 and was discussed as it was surprising [10]. Furthermore, *Using problem-solving activities* did not appear for the younger children in the baseline group. The ability to remember and express themselves can vary depending on age and individual variation because of social, emotional and cognitive development [21, 22] and individual differences may explain why these subcategories did not occur for younger children in the baseline group.

The present study consisted of several techniques for preparation and distraction in a complex environment, making the evaluation complicated. Interventions for reduction of distress during the child’s RT are not as extensively evaluated as non-pharmacological strategies (NPS) for needle-related procedures [23] where there now is a consensus on the efficacy of distraction and hypnosis for reduction of pain and distress in children during single minor procedures [24].

Children with cancer undergo repeated, painful and distressing procedures and several NPS used during cancer-related medical procedures are shown to reduce pain [25]. Psychological preparation and combined cognitive behavioral interventions for cancer-related procedures has been recommended, although there is still surprisingly little evidence for preparatory information [26]. Törnqvist, Månsson and Hallström [27], used an intervention similar to that of our study for children having magnetic resonance imaging and found it preferable to anesthesia or deep sedation.

Overall evaluations of NPS are mainly performed using quantitative methods such as self-reports, observational and physiological measures, but these do not always show group differences, and there is a lack of qualitative studies where children and adolescents describe their experiences and provide more nuanced understanding [28]. This is in line with the present study where the interviews provided the rich data and the quantitative measures gave less weight to the interpretation of results, partly also explained by a low number of children for statistical analysis.

The interviews revealed that the intervention suited younger children better than older ones. In particular they reported using the stuffed toy Hugo, and had suggestions for the tablet and the way they used the material for distraction. The children could make individual choices for distraction, which is emphasized as important for effective distraction and giving children a sense of control. They also reported appreciation of playing with Hugo and the CT/RT models, which is in line with other studies on the positive effect of playing with therapeutic toys [29]. The play provided them with an opportunity to process their experiences of undergoing RT both in advance and during the process, as most children receive RT for several weeks, and their siblings and friends also joined in the play. Distraction techniques are shown to be useful for children and adolescents of all ages though most of the studies are performed with children younger than 12 years [28].

Children from 11 years and older have greater cognitive skills, more understanding of complex situations and more elevated strategies to handle situations compared to younger children [21, 22]. There is a strong evidence of distraction being efficient for needle-related pain and distress [23]. Older children need more sophisticated distraction techniques, adapted to appropriate developmental stages, for when they have to deal with painful procedures [30]. There are few studies describing repeated daily distress for weeks as during RT. A tablet-based program, Pain buddy, was tested in a pilot study to enhance pain management in children aged 8 to 18 years undergoing cancer treatment and included cognitive and behavioral skills training [31]. Children reported using some non-pharmacological pain-management strategies such as positive self-talk, relaxation exercises, distraction techniques, breathing techniques and social support [31] comparable to what children described using in the present study and at baseline [10]. This is in line with secondary control or accommodative coping with efforts to adapt to stress, e.g. by positive thinking, distraction, and acceptance [32, 33]. HUGO for Teens had an application that served as a platform for sharing information. The older children reported that they did not use the information so much and required more integrative solutions. They found the visit to the RT room clarifying as a part of the preparation ahead of RT start; this was reported in the present study as well as at baseline [10]. According to the older children they need individualized information, strategies and support during RT. However, they can handle the situation and do not need the parents close by as the younger children do.

This research study has emphasized the importance of family-centered preparation. The parents of the children in the present study took an active part in preparing their children for RT by training them to lie motionless and practice with the mask and they participated throughout their children’s RT. The younger children played with the CT and RT models and chose the kind of distraction they wanted. The strength of the intervention in the present study was that it encouraged interaction within the family and provided an opportunity for siblings and peers to take part in what the child was going through. These findings are in line with other findings [34], that a family-centered preparation program (ADVANCE) was shown to be as effective as Midazolam in reducing children’s (2–10 years old) preoperative anxiety, thus meaning reduced stress within the family. The study by Fortier and co-authors [34] is one of few evaluating preparatory coping exercises with the family before going through a procedure where researchers dismantled what components of a multimodal family-centered preoperative preparation program were most effective. They found that practicing at home with the anesthesia mask, parental planning and use of distraction reduced the children’s preoperative anxiety the most [34]. By enabling and empowering children and their parents to have an active role, family-centered care can lead to safer, personalized and effective care and improved health-care experiences and further, a mutual confidential relationship can develop between child, family and staff members [35].

Family systems intervention practices are described where families with a child with cancer experienced a lessening of family suffering through therapeutic conversations [36]. Our reflection is that the family-systems intervention in the present project had similar effects. Family members cooperated, opened up to talk and listen to each other’s thoughts about the situation and that in turn created possibilities to find strategies to manage the situation of going through the RT.

Some methodological aspects need to be addressed. Although the design had an HCD approach including collaboration with families in the development of the intervention, some technical problems occurred and one child pointed out that the information about the radiation was not completely correct. Even though efforts were made to avoid this kind of problem a longer test period could have eliminated such issues. There were no significant differences found between groups regarding anxiety, neither from the children’s rating nor from parents’ proxy ratings of children’s anxiety. It is possible that a greater number of participants and less missing data could have shown statistical differences. When planning the project we assumed that both parents should rate their child’s anxiety at each study event although since parents share the duties in the family i.e. taking care of siblings usually only one of the parents followed the child to the RT.

The number of participants having anesthesia currently is already low, in this case only five in both groups, and is probably not a sensitive enough measurement method to display group differences. Earlier studies have shown less use of anesthesia because of good preparation and distraction [4, 5].

The FAS and VAS-A instruments are frequently used for assessing unpleasant experiences associated with single distressing and painful procedures in children. However, it is difficult to find instruments that fit this kind of study exactly, with an overall distress due to the cancer diagnosis and the nature of the repeated RT procedures with elements of habituation embedded in the process. The stress could probably be rated as constant through all kinds of procedures [37] and finding varying degrees of anxiety for the specific RT process may need more developed instruments or methods.

The credibility of the qualitative data was ensured by a heterogeneous sample regarding age and gender [38] and by the fact that the qualitative findings from the previous study [10] were confirmed in the present study. Similarities and differences among the children are somewhat dependent on different ages, development and maturity. The authors (GE, CÅB) who analyzed data have solid experience in analyzing qualitative data. Credibility was achieved through dialogue about the analysis among the authors [20]. The authors strived for an open mind to avoid interpretation not based on data. In the interviews, the children shared their experiences about RT, giving rich data, they made drawings and they offered proposals for improvements which make them trustworthy. Trustworthiness was achieved by choosing children with various experiences, genders and in different ages [20]. Authentic citations are provided [18]. To combine prior research findings in the deductive analysis with new findings from the inductive analysis strengthened the findings [19]. The transferability to similar contexts in Sweden or to a broader context may be possible. Healthcare professionals in similar surroundings may judge if it is transferable to their context.

### Recommendations/suggestions

This study provides several insights that could guide future design of research within the same field. The complex nature of this kind of intervention requires a strict protocol for checking treatment fidelity to intervention. This include pre-testing, training, monitoring of delivery of the intervention and a record of how the intervention was received by the children and the families [39]. Ethnographic methods involving parents and children in the development process was shown to be successful and is suitable for further development of the interventions. To train the parents in coping skills tailored to address their child’s anxiety before RT would strengthen a future intervention design similar to that created by Kain and co-authors [40], where they applied a web-based preparation program for children’s outpatient surgery. Clinical implications can be derived from the interviews with parents and children in the present study, revealing the extreme importance of organizing care in a family-centered way, especially for the younger children, and with respect given to adolescents’ needs regarding peers and integrity.

## Conclusion

The children of all the age groups experienced their interventions as positive. The strength of the intervention was that it encouraged interaction within the family and provided an opportunity for siblings and peers to take part in what the child was going through. The intervention was most appropriate for the younger children, who enjoyed the digital story, the stuffed animal and the opportunity to train with their parents. Younger and older children’s suggestions for more adapted and interactive solutions will provide a basis for development of the intervention. Future research on children’s experiences to interventions should be encouraged.

## Abbreviations

CNS: Central nervous system
CT: Computed tomography
FAS: Faces affective scale
HCD: Human centered design
RT: Radiotherapy
VAS-A: Visual analogue scale for anxiety
